# Sex differences in contextual pattern separation, neurogenesis, and functional connectivity within the limbic system

**DOI:** 10.1186/s13293-022-00450-2

**Published:** 2022-07-23

**Authors:** Shunya Yagi, Amanda Lee, Nadine Truter, Liisa A. M. Galea

**Affiliations:** 1 Graduate Program in Neuroscience, University of British Columbia, Vancouver, BC V6T 1Z3 Canada; 2 Department of Psychology, University of British Columbia, Vancouver, BC V6T 1Z4 Canada; 3 Djavad Mowafaghian Centre for Brain Health, University of British Columbia, 2215 Wesbrook Mall, Vancouver, BC V6T 1Z3 Canada

## Abstract

**Background:**

Females are more likely to present with anxiety disorders such as post-traumatic stress disorder (PTSD) compared to males, which are associated with disrupted hippocampal integrity. Sex differences in the structure and function of hippocampus exist. Here, we examined sex differences in contextual pattern separation, functional connectivity, and activation of new neurons during fear memory.

**Methods:**

Two-month-old male and female Sprague-Dawley rats were injected with the DNA synthesis markers, iododeoxyuridine (IdU) and chlorodeoxyuridine (CldU) 3 weeks and 4 weeks before perfusion, respectively. One week after CldU injection, the rats underwent a context discrimination task in which rats were placed in context A (shock) and context A’ (no shock) every day for 12 days. On the test day, rats were placed in the shock context (context A) to measure fear memory and expression of zif268, an immediate early gene across 16 different limbic and reward regions. Repeated-measures or factorial analysis of variance was conducted on our variables of interest. Pearson product-moment calculations and principal component analyses on zif268 expression across regions were also performed.

**Results:**

We found that females, but not males, showed contextual discrimination during the last days of training. On the test day, both sexes displayed similar levels of freezing, indicating equivalent fear memory for context A. Despite similar fear memory, males showed more positive correlations of zif268 activation between the limbic regions and the striatum, whereas females showed more negative correlations among these regions. Females showed greater activation of the frontal cortex, dorsal CA1, and 3-week-old adult-born dentate granular cells compared to males.

**Conclusions:**

These results highlight the importance of studying sex differences in fear memory and the contribution of adult neurogenesis to the neuronal network and may contribute to differences in susceptibility to fear-related disorders such as post-traumatic stress disorder.

**Highlights**

- Female rats, but not male rats, show faster discrimination during a contextual pattern separation task.
- Three-week-old adult-born neurons are more active in response to fear memory in females compared to males.
- Females had greater neural activation compared to males in the frontal cortex and dorsal CA1 region of the hippocampus in response to fear memory.
- Males and females show distinct patterns in functional connectivity for fear memory across limbic regions.
- Males have many positive correlations between activated new neurons of different ages between the dorsal and ventral hippocampus, while females show more correlations between activated new neurons and other limbic regions.

**Supplementary Information:**

The online version contains supplementary material available at 10.1186/s13293-022-00450-2.

## Introduction

Females are more likely to present with anxiety disorders such as post-traumatic stress disorder (PTSD) compared to males [1, 2], disorders which are associated with disrupted hippocampal integrity [3, 4]. The hippocampus plays important roles for pattern separation and pattern completion [5, 6]. Pattern separation refers to the process of forming distinct representations of similar inputs during memory encoding [6] and is a major component of episodic memory. Impairments in pattern separation are involved in overgeneralization of fear memory among patients with PTSD [4, 7].

Adult hippocampal neurogenesis is required for pattern separation and for stress resilience, as rodents with ablation of adult neurogenesis show impairments during pattern separation tasks and reduced stress resilience [8, 9]. Sex differences have been noted in the ability for pattern separation and the neurogenic response to pattern separation [10]. But curiously, these sex differences show either a male advantage or a female advantage in context discrimination tasks in rodents [11, 12] and in emotional episodic tasks in humans [13–15]. These differences in findings may be due to the availability of allocentric cues and egocentric cues in the context, as there are sex differences in preferential cue and strategy use in both humans [16] and rodents [17]. Curiously, new neurons are required for pattern separation in both males and females [8, 18], but pattern separation, using the delayed non-match to sample radial arm maze, increased hippocampal neurogenesis in male but not female rats [10]. Although no sex differences have been observed in activation of new neurons in response to spatial learning [10, 19], to our knowledge, no studies have examined possible sex differences in the activation of new neurons in response to fear memory after a contextual pattern separation task. Given that there are sex differences in the timing of maturation of new neurons in the hippocampus [20], it is important to determine whether different ages of neurons are active in response to fear memory after a contextual pattern separation task.

Studies demonstrate that hippocampus–amygdala–frontal cortex connectivity plays a critical role for long-term fear memory in humans [21, 22] and there are sex differences in the resting-state functional connectivity within this circuit [23]. In rodents, functional connectivity has been investigated via immediate early gene (IEG) mapping [24, 25]. IEGs, such as *zif268,* are genes that are rapidly induced in response to neuronal stimulation and IEG proteins play an important role in neural plasticity and memory [26–28]. Brain-wide IEG imaging in rodents can detect coordinated activation with high spatial resolution, which is useful to describe functional connectivity [25]. New neurons contribute to contextual fear memory [29], with younger new neurons more likely to play a critical role for pattern separation [8, 30]. However, there are no studies, to our knowledge, examining possible sex differences in patterns of activation with new neurons of different ages. Furthermore, it remains to be determined whether new neurons in the dentate gyrus (DG) are activated in a coordinated fashion with other brain regions, and whether sex modulates the functional connectivity of adult-born neurons during recall of fear memory.

Therefore, we examined sex differences in contextual pattern separation and functional connectivity among 16 different limbic and reward regions during fear memory retrieval. A fear-conditioning context discrimination task was used to assess sex differences in the ability to discriminate between two contexts in male and female rats. As younger new neurons contribute to pattern separation more so than older new neurons [30], and there are sex differences in the timing of maturation of new neurons [20], we capitalized on different methods to examine the activity of 2-week, 3-week or 4-week-old new neurons in response to fear memory. We used two different thymidine analogues that can be used in concert, along with an endogenous marker of immature neurons, to understand how different ages of new neurons responded to fear memory retrieval. Furthermore, we examined the activation of these different ages of new neurons in combination with IEG imaging and examined the coordinated neuronal activation of adult-born dentate granular cells (DGC)s with other brain regions. We hypothesized that there would be sex differences in context discrimination and activity of new neurons dependent on age of the new neuron. Furthermore, we also predicted that males and females would show distinct patterns of coordinated neuronal activation of different brain regions (hippocampus, amygdala, frontal cortex and striatum) during fear memory retrieval and that new neurons would show disparate patterns of functional connectivity between the sexes.

## Methods

### Subjects

Sixteen 8-week-old Sprague-Dawley rats (males: *n* = 8; females: *n* = 8) were purchased from Charles River Canada (St-Constant, QC, Canada). Rats were pair-housed in opaque polysulfone bins (432 mm × 264 mm × 324 mm) with paper towels, a single polycarbonate hut, virgin hardwood chip bedding, and free access to food and water. Males and females were housed in separate colony rooms that were maintained under a 12:12-h light/dark cycle (lights on at 07:00 h). All animals were handled every day for two minutes beginning one week after arrival for two weeks. All experiments were carried out in accordance with the Canadian Council for Animal Care guidelines and were approved by the animal care committee at the University of British Columbia. All efforts were made to reduce the number of animals used and their suffering during all procedures.

### Apparatus

**Fig. 1:**
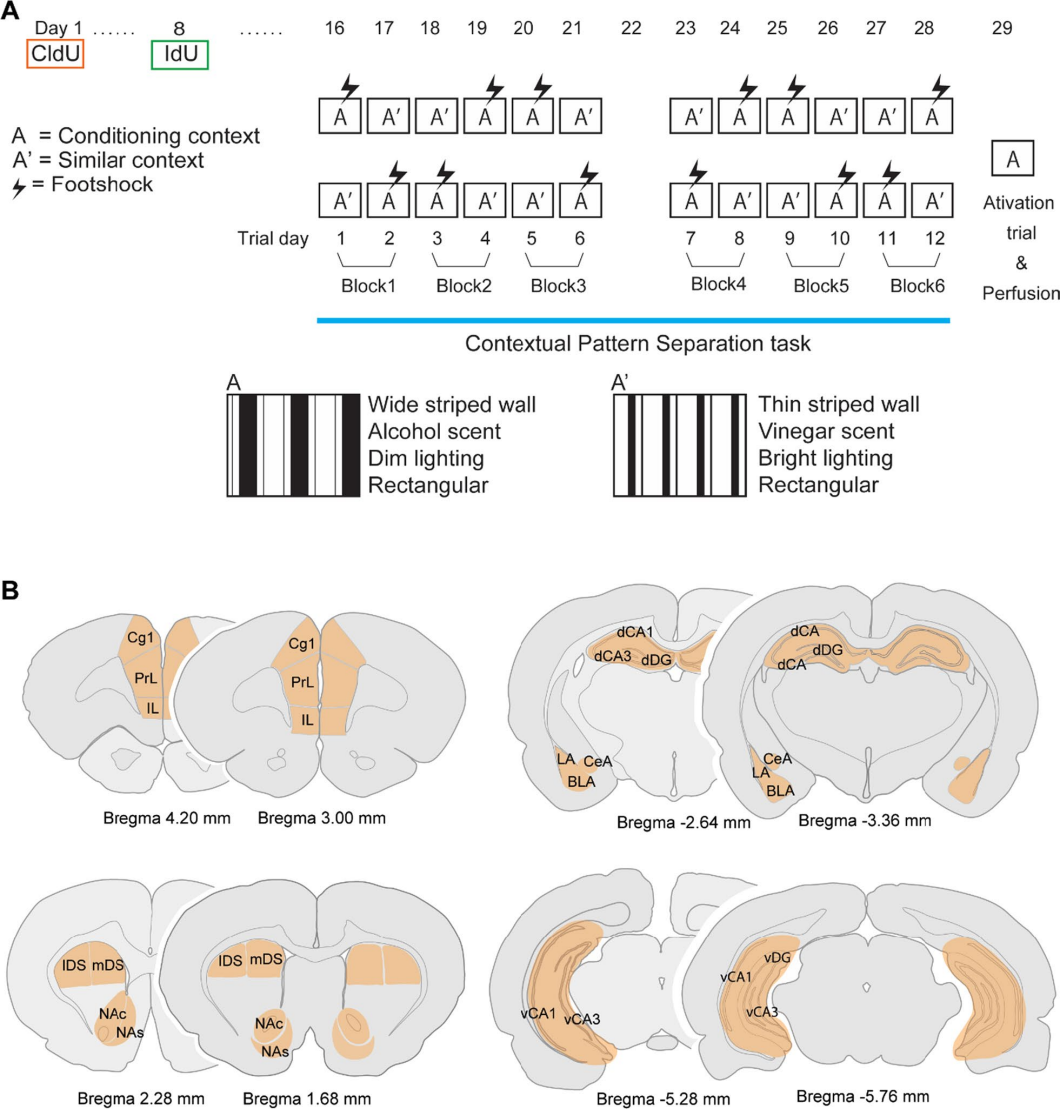
Experimental design. **A** Schematic illustration of experimental timeline: subjects received one intraperitoneal injection of 5-chloro-2'-deoxyuridine on Experimental Day 1 and one intraperitoneal injection of 5-iodo-2'-deoxyuridine on Experimental Day 8. Then, subjects were tested in the contextual pattern separation task for a total of 12 days (Experimental Day 16–28), followed by an activation trial in which the rats were placed in the context previously paired with shock but received no shock (Experimental Day 29). During the contextual pattern separation task, subjects were exposed to two different contexts each day; context A a shock-paired context (context paired with foot shocks) and context A’ a neutral context (context with no foot shock). **B** Brain regions that were examined for functional connectivity using zif268. *ACC* cingulate cortex (Cg1), *PrL* prelimbic cortex, *IL* infralimbic cortex, *lDS* lateral dorsal striatum, *mDS* medial dorsal striatum, *NAc* nucleus accumbens core, *NAs* nucleus accumbens shell, *LA* lateral amygdala, *BLA* basolateral amygdala, *CeA* central amygdala, *dDG* dorsal dentate gyrus, *vDG* ventral dentate gyrus, *dCA1* dorsal cornu ammonis 1, *vCA1* ventral cornu ammonis 1, *dCA3* dorsal cornu ammonis 3, *vCA3* ventral cornu ammonis 3

Behavioral testing for all experiments was conducted in four operant chambers (30.5 × 24 × 21 cm; Med-Associates, St Albans, VT) enclosed in sound-attenuating boxes. The boxes were equipped with a fan to provide ventilation and to mask extraneous noise. All behaviors were monitored and recorded by a single video camera mounted on the ceiling of each box. The chambers were equipped with a single 100-mA houselight located in the top center of a wall and the chamber floor consisted of 23 metal grid bars (0.4 cm in diameter) that ran parallel to the shorter wall of the chamber, which connected to a shock generator. Two chambers had wide vertical black (18 mm width) and white (12 mm width) stripe patterns on the walls and wiped with vinegar before and after each animal. The other two chambers had narrow vertical black (12 mm width) and white (12 mm width) stripe patterns on the walls and wiped with 70% isopropanol before and after each animal (see Fig. 1). All the chambers were connected to a computer through a digital interface that recorded all experimental settings.

### Procedures

#### Experimental timeline

Subjects received one injection of 5-chloro-2'-deoxyuridine (CldU:171 mg/kg; intraperitoneal (i.p.), MP Biomedicals, Santa Ana, CA, USA) on Experimental Day 1 and one injection of 5-iodo-2'-deoxyuridine (IdU: 56.75 mg/kg; i.p., Cayman Chemical, Ann Arbor, MI, USA) on Experimental Day 8, thus ensuring that we examined 4 week old and 3 week old cells, respectively. A previous study demonstrates that adult-born neurons reach the full maturation four weeks after BrdU injection in rats [31], the present study examined zif268 expression of 4-week-old neurons as activation of fully matured neurons. Thymidine analogs, IdU and CldU, incorporate into DNA during synthesis phase of cell proliferation, which can be distinguished from one another using respective antibodies [32–35]. Subjects were tested in the contextual pattern separation task (modified from [36]) for 12 days (Experimental Days 16–28, which are referred to as Trial Days 1–12), followed by a day of activation test trial that is described below (Experimental Day 29; see Fig. 1A).

#### Behavioral testing for contextual pattern separation

Subjects were exposed daily for 5 min each to two different contexts (4–5 h interval between contexts), a shock-paired context (Context A) and a neutral context (Context A’), for a total of 12 days. The contexts for Context A trials and Context A’ trials were counterbalanced across subjects and remained the same for each subject throughout the entire experiment. During the shock-paired trial in Context A, subjects were allowed to explore the chamber for three minutes followed by three 1-s foot shocks (0.6 mA) with 30-s intervals between each shock. The subjects returned to their home cage one minute after the third shock. During the neural trial in Context A’, the subjects explored a different context from Context A for five minutes without receiving a foot shock and returned to their home cage. The order of two contexts that subjects were exposed each day for the first 6 days followed AA’–A’A–A’A–AA’–AA’–A’A design, and the order was reversed for the remaining days [36] (see Fig. 1A).

The duration of freezing during the first 3 min of each trial (prior to any shocks) was examined as the conditioned fear response, and the percentage of freezing was calculated by dividing the duration of freezing by 180 s. A discrimination index (DI) was calculated with the following formula on the last two days of training:

$$\rm{DI}=\frac{(\rm{freezing \, time \, in \, Context \, A }-\rm{ freezing \, time \, in \, Context \, A^{\prime}})}{(\rm{freezing \, time \, in \, Context \, A }+\rm{ freezing \, time \, in \, Context \, A^{\prime}})}.$$

As a previous study found sex differences in darting, an active fear response, in a cued fear-conditioning task [37], darting behavior was also recorded.

#### Activation trial and perfusion

On the day after Training Day 12, the Activation Test Trial was conducted to examine fear memory. Subjects were exposed to the Context A for 5 min without a foot shock and returned to their home cage. Video recordings were analyzed for active fear behavior (darting), passive fear behavior (freezing), or other behaviors (rearing, grooming and non-specific behaviors; see Additional file 1). However, no darting in our paradigm was observed. Ninety minutes after the Activation trial, subjects were administered an overdose of sodium pentobarbital (500 mg/kg, i.p.) and perfused transcardially with 60 mL of 0.9% saline followed by 120 mL of 4% paraformaldehyde (Sigma‐Aldrich).

### Tissue processing

Extracted brains were postfixed in 4% paraformaldehyde overnight, then transferred to 30% sucrose (Fisher Scientific, Ottawa, ON, Canada) solution for cryoprotection and remained in the solution until sectioning. Brains were sliced into 30-μm coronal sections using a Leica SM2000R microtome (Richmond Hill, ON, Canada). Sections were collected in series of 10 throughout the entire rostral‐caudal extent of the forebrain (Bregma 5.64 to − 7.56 mm) and stored in antifreeze solution consisting of ethylene glycol, glycerol, and 0.1 M PBS at − 20 °C.

### Immunohistochemistry

Brain tissue was double-stained for the immature neuronal protein, doublecortin (DCX), and the immediate early gene, zif268 (see details of antibodies and reagents used in Additional file 1: Tables S1 and S2). A majority (70% or more) of adult-born granule cells express DCX within 24 h after mitosis for up to two weeks, with maximal expression at 4 days after mitosis, and DCX expression is rapidly reduced 3 weeks after mitosis (less than 20%) in both male and female rats [20, 31, 38]. Therefore, we used DCX to examine a cell population of new neurons that were larger 2 weeks old or younger. In addition, tissue was triple-stained for IdU, CldU, and zif268 to examine neural activation of 3-week-old (IdU) cells and 4-week-old (CldU) cells in the dentate gyrus.

#### Doublecortin/zif268 double labeling

Free-floating sections were prewashed three times for 10 min with 0.1 M Tris buffer saline (TBS; Sigma-Aldrich, Oakville, ON, Canada). Sections were then incubated in a primary antibody solution containing 1:500 rabbit anti-zif268 (Santa Cruz Biotechnology, Dallas, TX, USA), 1:500 goat anti-doublecortin (Santa Cruz Biotechnology, Dallas, TX, USA) 0.3% Triton-X (Sigma-Aldrich) and 3% normal donkey serum (NDS; MilliporeSigma, Burlington, MA, USA) in 0.1 M TBS for 24 h at 4 °C. Sections were washed three times for 10 min in TBS and a further incubation of sections commenced in a secondary antibody solution containing 1:500 donkey anti-rabbit ALEXA 594 (Invitrogen, Burlington, ON, Canada), 1:500 donkey anti-goat ALEXA 488 (Invitrogen, Burlington, ON, Canada), 3% NDS and 0.3% Triton-X in 0.1 M TBS for 24 h at 4 °C. Following three final rinses with TBS, the sections were mounted onto microscope slides and cover-slipped with PVA DABCO.

#### IdU/CldU/zif268 triple labeling

Two different thymidine analogues (CldU and IdU) were visualized with CldU-specific (rat monoclonal, clone BU1/75) and IdU-specific (mouse monoclonal, clone B44) antibodies [39], coupled with labeling using the immediate early gene, zif268 antibody (rabbit polyclonal). Briefly our protocol was as follows: free-floating sections were prewashed three times for 10 min with 0.1 M TBS. Sections were then incubated in a primary antibody solution containing 1:500 rabbit anti-zif268 (Santa Cruz Biotechnology, Dallas, TX, USA), 0.3% Triton-X (Sigma-Aldrich) and 3% NDS in 0.1 M TBS for 24 h at 4 °C. Next, sections were incubated in a secondary antibody solution containing 1:250 donkey anti-rabbit ALEXA 647 (Invitrogen, Burlington, ON, Canada), 0.3% Triton-X, and 3% NDS in 0.1 M TBS, for 18 h at 4 °C. After being rinsed three times for 10 min with TBS, sections were washed with 4% paraformaldehyde for 10 min, and rinsed twice in 0.9% NaCl for 10 min, followed by incubation in 2 N HCl (Fisher Scientific, Waltham, Massachusetts, USA) for 30 min at 37 °C. Sections were then rinsed three times in TBS for 10 min each and incubated in a CldU primary antibody solution consisting of 1:1000 rat anti-BrdU (BU1/75; Abcam; Toronto, ON, Canada), 3% NDS, and 0.3% Triton-X in 0.1 M TBS for 24 h at 4 °C. Sections were then incubated in an IdU primary antibody solution consisting of 1:500 mouse anti-BrdU (B44; BD Biosciences, San Jose, CA, USA), 0.3% NDS, and 0.3% Triton-X in 0.1 M TBS for 24 h at 4 °C. Sections were then washed twice for 10 min each in a high stringency wash solution consisting of 32 mM Tris buffer, 50 mM NaCl and 0.5% tween (pH 8.0) at 37 °C. Following three washes in TBS, sections were incubated in a secondary antibody solution containing 1:500 donkey anti-rat ALEXA 594 (Invitrogen, Burlington, ON, Canada), 1:500 donkey anti-mouse ALEXA 488 (Invitrogen, Burlington, ON, Canada), 3% NDS and 0.3% Triton-X in 0.1 M TBS for 24 h at 4 °C. Following three final rinses with TBS, the sections were mounted onto microscope slides and cover-slipped with PVA DABCO.

### Cell counting

All counting was conducted by an experimenter blind to the group assignment of each animal using an Olympus FV1000 confocal microscope and/or Zeiss Axio Scan.Z1 (Carl Zeiss Microscopy, Thornwood, NY, USA). Density of immunoreactive cells was calculated by dividing the total immunoreactive (ir) cells by volume (mm^3^) of the corresponding region. Volume estimates were calculated by multiplying the summed areas by thickness of sections (0.03 mm, using Cavalieri’s principle [40]). Area measurements for the region of interest were obtained using digitized images on Zen 3.0 software (blue edition; Carl Zeiss Microscopy, Thornwood, NY, USA).

Brain regions were defined according to a standard rat brain atlas [41]. Location of immunoreactive cells in the dorsal or ventral hippocampus was examined using the criterion defined by Banasr et al. [42] with sections 7.20–4.48 mm from the interaural line (Bregma − 1.80 to − 4.52 mm) defined as dorsal and sections 4.48–2.20 mm from the interaural line (Bregma − 4.52 to − 6.80 mm) as ventral [42]. Cells were counted separately in each region because the different regions are associated with different functions (reviewed in [43]) and different maturation timelines of neurogenesis [13, 20]. The dorsal hippocampus is associated with spatial reference memory, whereas the ventral hippocampus is associated with working memory, stress and anxiety [14, 15].

#### IdU and CldU counting

Thymidine analogue immunoreactive (IdU-ir and CldU-ir) cells were counted under a 40 × objective lens using Olympus FV1000 confocal microscopy. Every 20th section of the granule cell layer (GCL) that includes the subgranular zone (SGZ) was counted. The SGZ was defined as a narrow layer of cells within 30 μm (equivalent to the width of three granule cell bodies) away from the innermost edge of GCL (Redila and Christie, 2006).

The percentages of IdU/zif268 and CldU/zif268-ir cells were obtained by randomly selecting 200 IdU-ir or 200 CldU-ir cells (100 cells from dorsal and 100 cells from ventral DG) and calculating the percentage of cells that were double-labeled with zif268 under a 40 × objective lens using Olympus FV1000 confocal microscopy. Density of DCX/zif268-ir, IdU/zif268-ir or CldU/zif268-ir cells were calculated by multiplying the density of IdU-ir or CldU-ir cells by the percentage of double-labeled cells.

#### Doublecortin counting

Doublecortin immunoreactive (DCX-ir) cells were counted on digitized images on Zen 3.0 software (blue edition). Photomicrographs were taken from four dorsal and four ventral hippocampi using a ZEISS Axio Scan.Z1 slidescanner with a 40 × objective lens. The percentages of DCX/zif268-ir cells were obtained by randomly selecting 200 DCX-ir cells (100 cells from dorsal and 100 cells from ventral DG) and calculating the percentage of cells that were double-labeled with zif268 on Zen 3.0 software. Density of DCX/zif268-ir cells were calculated by multiplying the density of DCX-ir cells by the percentage of DCX/zif268-ir cells.

*Estrous cycle determination*. Vaginal cells suspended in water were obtained using a glass pipette, transferred onto microscope slides, stained with Cresyl Violet (Sigma), and analyzed using a 20 × objective. Proestrus stage was determined when 70% of the cells were nucleated epithelial cells [44].

#### zif268 counting

Photomicrographs of coronal sections containing the frontal cortex, amygdala, hippocampus, dorsal striatum, nucleus accumbens were obtained from ZEISS Axio Scan.Z1 slidescanner with a 20 × objective lens (four images from each region of interest: see Fig. 1B). Zif268-ir cells in the infralimbic cortex (IL), prelimbic cortex (PrL), anterior cingulate cortex (ACC: Cg1), medial part of dorsal striatum (mDS), lateral part of dorsal striatum (lDS), nucleus accumbens core (NAc), nucleus accumbens shell (NAs), central nucleus of amygdala (CeA), basolateral nucleus of the amygdala (BLA), lateral nucleus of the amygdala (LA), dorsal(*d*) hippocampus (dCA1, dCA3, dDG) and ventral(v) hippocampus (vCA1, vCA3, vDG) were counted automatically from the digitized images using a code developed by JEJS (see [20] for details) on MATLAB (MathWorks; Natick, Massachusetts, USA).

### Estrous cycle determination

Daily lavage samples were taken from all females after behavioral procedures (see Additional file 1: Methods section). Estrous cycle determination was done as the estrous cycle stage can affect long-term potentiation and IEG expression in the hippocampus [45, 46]. There was one female in the proestorus stage during the Activation Trial and thus, estrous cycle phase was used as a covariate for all analyses.

### Statistical analyses

All analyses were conducted using Statistica (Statsoft Tulsa, OK) unless otherwise stated, and significance level was set at *α* = 0.05. Repeated-measures or factorial analysis of variance (ANOVA), with sex (male and female) as between-subject variables were conducted on our variables of interest (freezing, zif268 expression). Post-hoc tests used the Newman–Keuls procedure.* A priori* comparisons were subjected to Bonferroni corrections. Effect sizes are given with Cohen’s *d* or partial *η*^2^. Pearson product-moment calculations and principal component analyses on zif268 expression across regions were also performed.

The percentage of freezing during the Context A trials and Context A’ trials in the contextual pattern separation task was analyzed using repeated-measures analysis of variance (ANOVA), with sex (male and female) as between-subject variables and context (Context A and Context A’) and trial day (1st–12th day) as within-subject factors. The discrimination index of the last trial block and percentage of freezing during the activation trial were analyzed using one-way ANOVA with sex as between-subject variable. The density of adult-born cells (DCX-ir, IdU-ir or CldU-ir cells) and those double-labeled with zif268 in the dentate gyrus were each analyzed using repeated-measures ANOVA with sex as between-subject variable and region (dorsal and ventral) as within-subject variable. The density of zif268-ir cells in each region (frontal cortex, dorsal striatum, nucleus accumbens, amygdala) was analyzed separately using repeated-measures ANOVA with sex as between-subject variables and subregions (frontal cortex: IL, PrL, ACC; dorsal striatum: lateral, medial; nucleus accumbens: core, shell; amygdala: central, lateral, basal; hippocampus: dorsal and ventral CA1, CA3 and DG) as within-subject variables.

Pearson product–moment correlations between the percentage of freezing and the density of zif268-ir cells were calculated in the regions of interest. For functional connectivity, Pearson product–moment correlations were calculated with the density of zif268-ir cells between each brain region. To examine the functional connectivity of adult-born cells in the dentate gyrus with the other brain regions, correlations were also calculated between the density of IdU/zif268-ir, CldU/zif268-ir or DCX/zif268-ir cells and the density of zif268-ir cells in each region. Inter-regional correlations were compared between the two sexes (male and female) using the single-sided observed Fisher z-test statistic.

Principal component analyses were conducted to assess brain networks that explain variances of zif268-ir cell density in the regions of interest. PCA data analyses were conducted using Statistica and R (3.4.3) statistical analysis software with the “FactoMineR” package. Horn’s parallel analysis was used to determine which component factors were retained for further analyses [47]. Horn’s parallel analysis was conducted using R (3.4.3) statistical analysis software with the “psych” package. Following the principal component analysis, a repeated-measures ANOVA was conducted with the principal component factor scores (1st, 2nd, 3rd) as the within-subject variable and sex (male, female) as the between-subject variable.

## Results

### Females, but not males, discriminated shock-paired contexts from neutral contexts

**Fig. 2:**
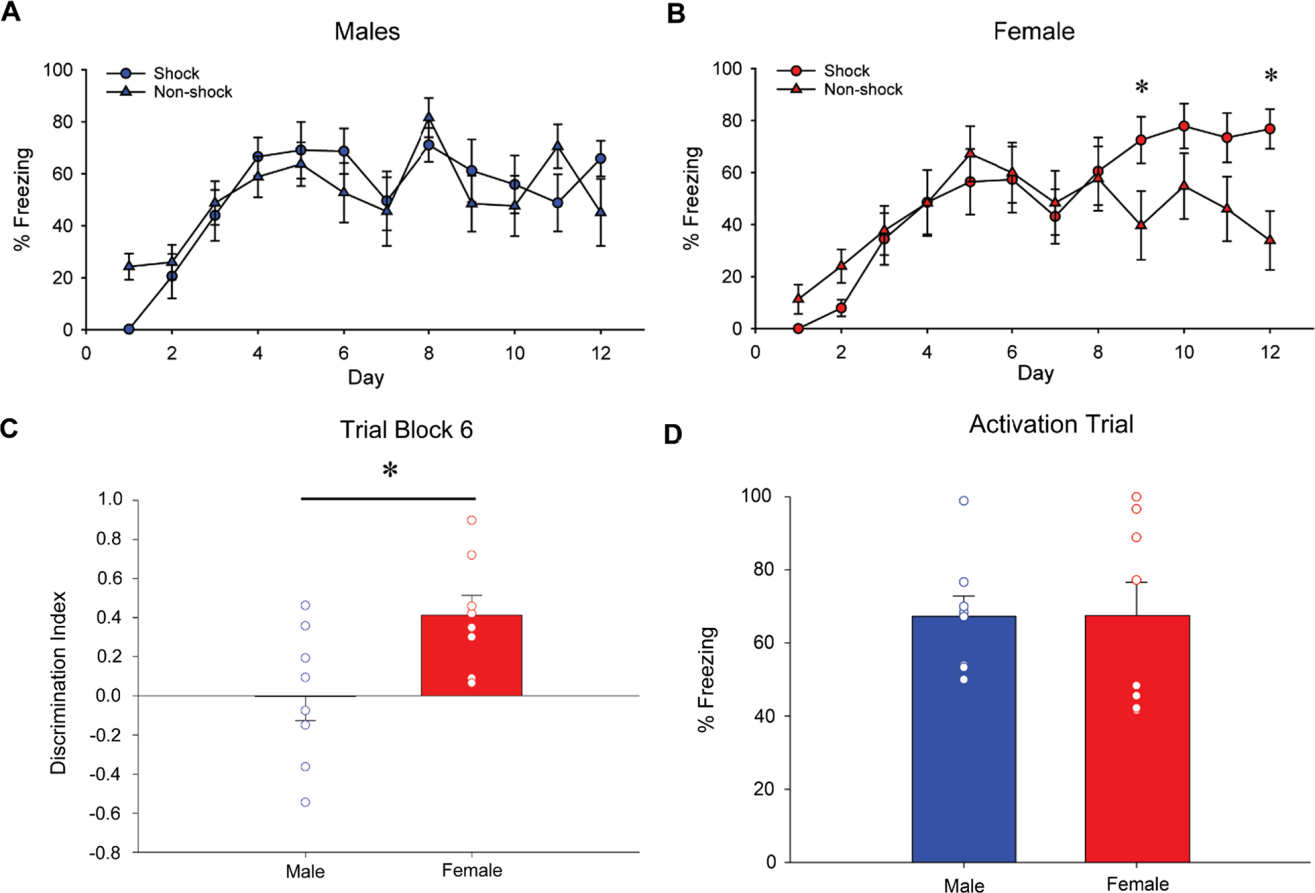
Sex differences in contextual fear discrimination. Mean (± SEM) percentage of freezing in Context A (shock) and Context A’ (non-shock) in males (**A**) and females (**B**). Mean (± SEM) discrimination index of the trial block 6 (Trial Day 11 + 12) (**C**). Mean (± SEM) percentage of freezing in males and females during the activation trial on Day 29 (**D**). Females exhibited significantly greater percentage of freezing in the shock-paired context (Context A) than in the neutral context (Context A’) on Trial Day 9 and Trial Day 12, whereas there was no significant difference in percentage of freezing between the two contexts in any days in males. Furthermore, females showed greater discrimination based on the index on the last trial block (Trial Day 11 + 12) compared to males. There was no significant sex difference in the percentage of freezing during the Activation Trial. *Indicates *p* < 0.05

Male and female rats were exposed to 12 days of the contextual pattern separation task to examine the ability for discriminating a shock-paired context from a neutral context. Females exhibited a significantly greater percentage of freezing in the shock-paired context (Context A) than in the neutral context (Context A’) on two days: Trial Day 9 (*p* < 0.02, Cohen’s *d* = 1.114), and Trial Day 12 (*p* < 0.0001, Cohen’s *d* = 1.696), whereas there were no significant differences between the contexts on any day in males (Fig. 2A and B; interaction effect of sex by day by context [*F*(11, 154) = 2.25, *p* = 0.014, *η*_p_^2^ = 0.139)]). There was also a significant interaction effect of day by context [*F*(11, 154) = 6.26, *p* < 0.0001, *η*_p_^2^ = 0.309] and a main effect of day [*F*(11, 154) = 21.04, *p* < 0.0001, *η*_p_^2^ = 0.600]. Consistent with these findings, the discrimination index (DI) of the last two days (Trial Day 11 and 12 or Block 6) indicated that females showed a greater DI compared to males [*F*(1, 14) = 5.81, *p* = 0.030, Cohen’s *d* = 1.205; Fig. 2C].

On the activation trial day, all subjects were exposed to the conditioning context A (on the day after the 12 training Trials) without any shock to assess fear memory. There was no significant sex difference in the percentage of freezing during the activation trial (*p* = 0.932, Cohen’s *d* = 0.045; Fig. 2D), indicating the memory strength for the shock-paired context was equivalent between the sexes, despite the better discrimination learning in females. As noted earlier, no darting behavior was observed throughout the experiment.

### There were more 4-week-old cells in the dorsal compared to the ventral dentate gyrus

IdU and CldU were injected 3 weeks and 4 weeks, respectively, before perfusion. The density of CldU-ir cells was greater in the dDG compared to vDG [main effect of region: *F*(1, 11) = 6.50, *p* = 0.027, Cohen’s *d* = 1.104; see Additional file 1: Fig. S1]. There were no other significant main or interaction effects on the density of DCX-ir, CldU-ir or IdU-ir cells (*p*’s > 0.283).

### Females had a greater percentage of IdU/zif268-ir cells in the dentate gyrus compared to males

**Fig. 3:**
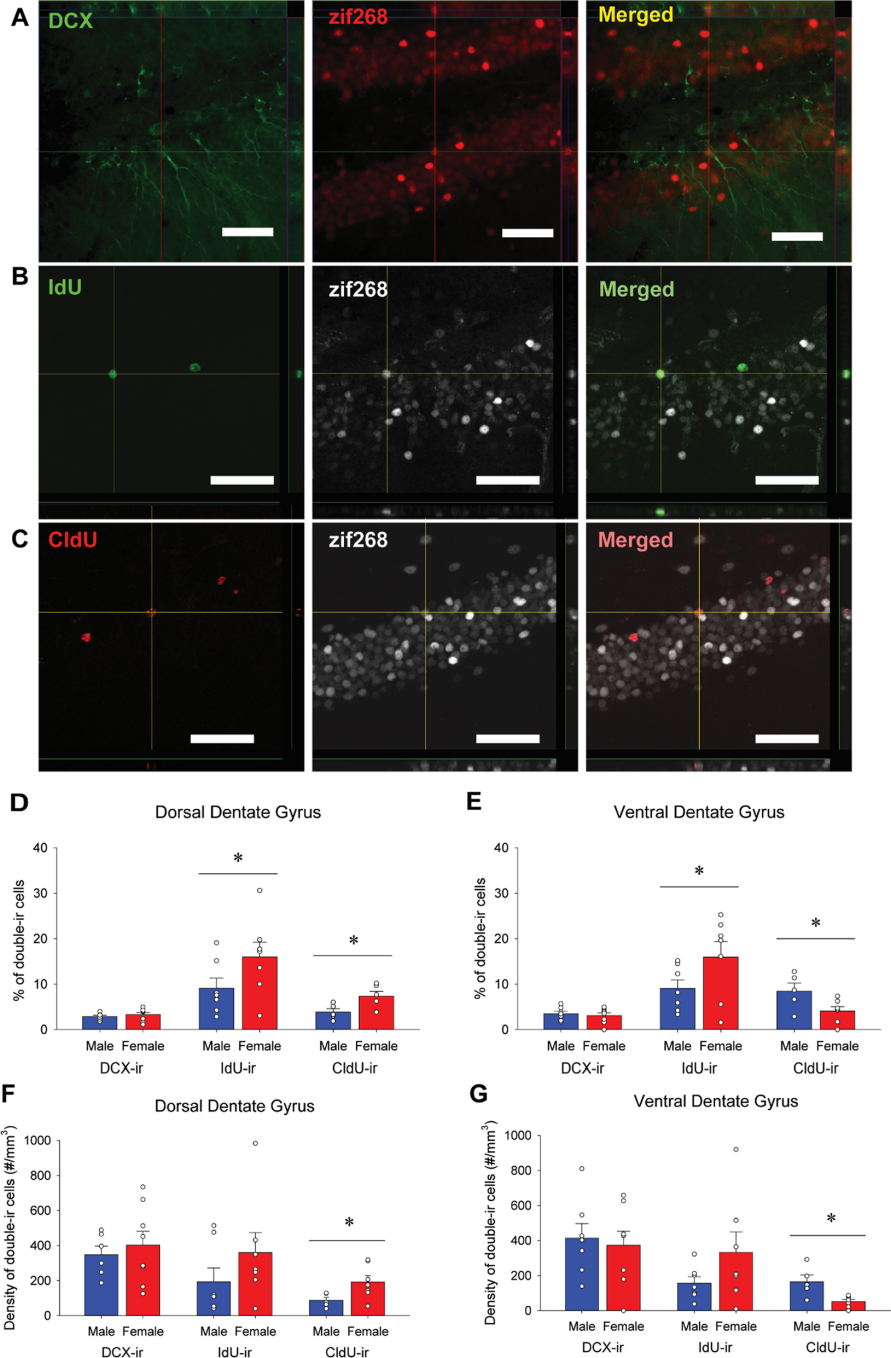
Sex differences in zif268 activation of dentate adult-born cells. Photomicrographs of doublecortin-immunoreactive (ir) cells (DCX-ir: green) and zif268-ir cells (red) were taken under Zeiss Axio Scan.Z1 with a 40 × objective lens (**E**). Photomicrographs of IdU-ir cells (green) and zif268-ir cells (white) were taken under Olympus FV1000 confocal microscope with a 40 × objective lens (**F**). Photomicrographs of CldU-ir cells (red) and zif268-ir (white) cells were taken under Olympus FV1000 confocal microscope with a 40 × objective lens (**G**). Scale bars indicate 50 μm. Mean (± SEM) percentage of double-labeled cells in the dorsal (**H**) and ventral (**I**) dentate gyrus. Females, compared to males, had greater percentage of IdU/zif268-ir cells in the dorsal and ventral dentate gyrus, whereas there was a significant interaction effect of sex by region for the percentage of CldU/zif268-ir cells, with females having a greater percentage of CldU/zif268 in the dorsal dentate gyrus but males having a greater percentage of CldU/zif268-ir in the ventral dentate gyrus. Mean (± SEM) density of double-labeled cells in the dorsal (**J**) and ventral (**K**) dentate gyrus. There was a significant interaction effect of sex by region for the density of CldU/zif268-ir cells, with females having a greater density of CldU/zif268-ir in the dorsal dentate gyrus but males having a greater density in of CldU/zif268-ir in the ventral dentate gyrus. *Indicates *p* < 0.05

The percentage of DCX-ir, IdU-ir, or CldU-ir cells that were double-labeled with zif268 was measured to examine neural activation of adult-born cells in the DG (Fig. 3A–C). Females, compared to males, had a greater percentage of IdU/zif268-ir cells in the DG [main effect of sex: *F*(1, 12) = 4.59, *p* = 0.05, Cohen’s *d* = 1.539; Fig. 3D and E] but no other main or interaction effects for IdU/zif268-ir cells. Females, compared to males, had greater percentage of CldU/zif268-ir cells in the dDG (*p* = 0.047, Cohen’s *d* = 1.538), whereas, males, compared to females, had greater percentage of CldU/zif268-ir cells in the vDG (*p* = 0.015, Cohen’s *d* = 1.317) [interaction effect of region by sex: *F*(1, 10) = 19.53, *p* = 0.001, *η*_p_^2^ = 0.661; Fig. 3D and E]. There were no significant main effects (all *p*’s > 0.46) for CldU/zif268-ir cells. There were no significant effects of DCX/zif268-ir cells (*p*’s > 0.66).

Along with the percentage of double-labeled cells we also examined the density of DCX-ir, IdU-ir, and CldU-ir cells that were double-labeled with zif268. Females, compared to males, had greater density of CldU/zif268-ir cells in the dDG (*p* = 0.033), whereas males, compared to females, had greater density of CldU/zif268-ir cells in the vDG (*p* = 0.023) [interaction effect of region by sex: *F*(1, 10) = 19.34, *p* = 0.001, *η*_p_^2^ = 0.659; Fig. 3F and G]. There were no main or interaction effects for the density of IdU/zif268-ir cells or DCX/zif268-ir cells (all *p*’s > 0.106).

### Females had greater zif268 immunoreactivity than males in the frontal cortex and dorsal CA1 region of the hippocampus in response to a shocked-paired context

**Fig. 4:**
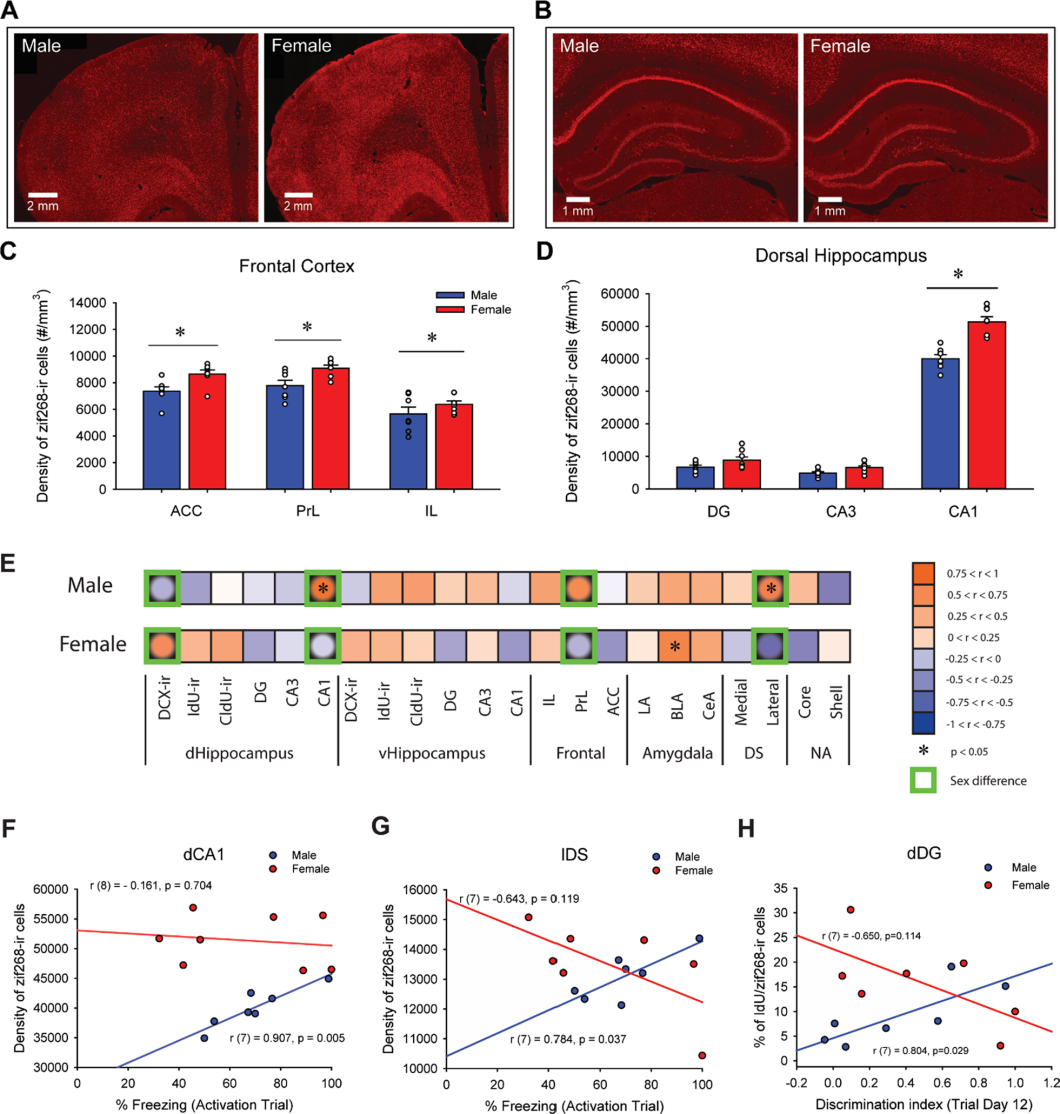
Sex differences in zif268 activation in the brain, and correlations with fear response. Photomicrographs of zif268 immunoreactivity in the frontal cortex (**A**) and in the dorsal hippocampus (**B**) in male (left) and female rats (right). Mean (± SEM) density of zif268-ir cells in the frontal cortex (**C**) and in the dorsal hippocampus (**D**). Females, compared to males, had greater density of zif268-ir cells in the anterior cingulate cortex (ACC), in the prelimbic cortex (PrL) and in the dorsal CA1. *Indicates *p* < 0.05. Heat maps generated based on correlations coefficients between the amount of freezing and activated cells (zif268-ir) in different regions of the limbic system (**E**). Males and females had significant correlations of zif268-ir cell density in different brain regions with the percentage of freezing (*indicates correlations with *p* < 0.05). There were significant sex differences in the correlations between the percentage of freezing and the density of zif268-ir cells in four different brain regions (Green boxes indicates significant sex differences with *p* < 0.05). Scatter plots for correlations between the percentage of freezing and the density of zif268-ir cells in the dorsal CA1 (dCA1) (**F**) and in the lateral dorsal striatum (lDS) (**G**), and correlations between the discrimination index on the last trial day and the percentage of IdU/zif268-ir cells in the dorsal dentate gyrus (**H**) in males (blue) and females (red)

The density of zif268-ir cells was measured to examine neural activation in subregions of the frontal cortex, dorsal striatum, nucleus accumbens, hippocampus and amygdala in response to exposure to the shocked-paired context. Females, compared to males, showed greater density of zif268-ir cells across the different regions of the frontal cortex [main effect of sex: *F*(1, 12) = 10.14, *p* = 0.008, *η*_p_^2^ = 0.458], as well there was greater density of zif268-ir cells in the ACC and PrL compared to IL [main effect of subregion: *F*(2, 24) = 34.40, *p* < 0.001, *η*_p_^2^ = 0.741; post hoc: all *p*’s < 0.001; Fig. 4C].

Furthermore, females showed greater density of zif268-ir cells in the dorsal CA1 compared to males [*p* < 0.001, Cohen’s *d* = 2.957; interaction effect of sex by subregion by dorsoventral axis: *F*(2, 26) = 17.56, *p* < 0.001; Fig. 4D]. In both males and females, the density of zif268-ir cells in the dCA1 is greater than dDG and dCA3, and the density of zif268-ir cells in the vCA1 is greater than vCA3 (all *p*’s < 0.01). There were also significant interaction effects of sex by subregion [*F*(2, 26) = 10.36, *p* < 0.001, *η*_p_^2^ = 0.444], sex by dorsoventral axis [*F*(1, 13) = 10.82, *p* = 0.006, *η*_p_^2^ = 0.454] and subregion by dorsoventral axis [*F*(2, 26) = 1028.60, *p* < 0.001, *η*_p_^2^ = 0.988], and main effects of sex [*F*(1, 13) = 21.53, *p* < 0.001, *η*_p_^2^ = 0.624], subregion [*F*(2, 26) = 819.06, *p* < 0.001, *η*_p_^2^ = 0.984] and dorsoventral axis [*F*(1, 13) = 690.17, *p* < 0.001, *η*_p_^2^ = 0.982].

There were no significant main or interaction effects involving sex in activation in the amygdala, striatum or the nucleus accumbens, but there were significant regional differences within these areas. The lDS had greater density of zif268-ir cells compared to the mDS [main effect of subregion: *F*(1, 12) = 6.88, *p* = 0.022, *η*_p_^2^ = 0.364; Additional file 1: Fig. S2A]. In the amygdala, the LA had greater density of zif268-ir cells compared to the CeA (*p* = 0.007) and the BLA (*p* < 0.001), and the CeA had greater density of zif268-ir cells compared to the BLA (*p* < 0.001) [main effect of subregion: *F*(2, 24) = 28.45, *p* < 0.001, *η*_p_^2^ = 0.703; Additional file 1: Fig. S2B]. In the nucleus accumbens, the density of zif268-ir cells was significantly greater in the shell compared to the core [main effect of subregion: *F*(1, 12) = 22.22, *p* < 0.001, *η*_p_^2^ = 0.649; Additional file 1: Fig. S2C]. There were no significant main or interaction effects of sex for the density of zif268-ir cells in any of these regions (P > 0.111).

### The density of zif268-ir cells in the dorsal CA1 was positively correlated with amount of freezing during memory recall in males, but not in females

Pearson product–moment correlations were calculated between the percentage of freezing during the activation trial and the density of zif268-ir cells in the 16 brain regions and the six different populations of adult-born cells (dorsal or ventral DCX/IdU/CldU co-expressing zif268; see Fig. 4E). There was a significant positive correlation between the density of zif268-ir cells and the percentage of freezing in males in the dCA1 [*r* (7) = 0.907, *p* = 0.005; Fig. 4F]. There were no other significant correlations between the density of zif268-ir cells and the percentage of freezing during the activation trial after Bonferroni corrections (*p*’s > 0.037). Sex differences in the correlations were noted, with males having positive correlations and females having negative correlations, between the percentage of freezing and the density of zif268 cells in the dDG (*p* = 0.041), dCA1 (*p* = 0.006; Fig. 4F), PrL (*p* = 0.040) and lDS (*p* = 0.005; Fig. 4G). See Additional file 1: Results for details.

### Neural activation of adult-born cells in the dentate gyrus was associated with the ability for pattern separation in males but not females

Pearson product–moment correlations were calculated between the percentage of DCX, IdU or CldU-ir cells that were double-labeled with zif268 and the discrimination index (DI) on the last trial day. The percentage of IdU-ir cells that were double-labeled with zif268 in the dDG was significantly positively correlated with DI in males [*r*(7) = 0.804, *p* = 0.029], but not in females [*r*(7) = − 0.650, *p* = 0.114; Fig. 4H], which was significantly different between the sexes (*p* = 0.004). There were no other significant correlations between the percentage of DCX, IdU or CldU-ir cells that were double-labeled with zif268 and the DI (all *p*’s > 0.127).

### Males and females showed distinct patterns of significant inter-regional correlations of the density of zif268-ir cells

**Fig. 5:**
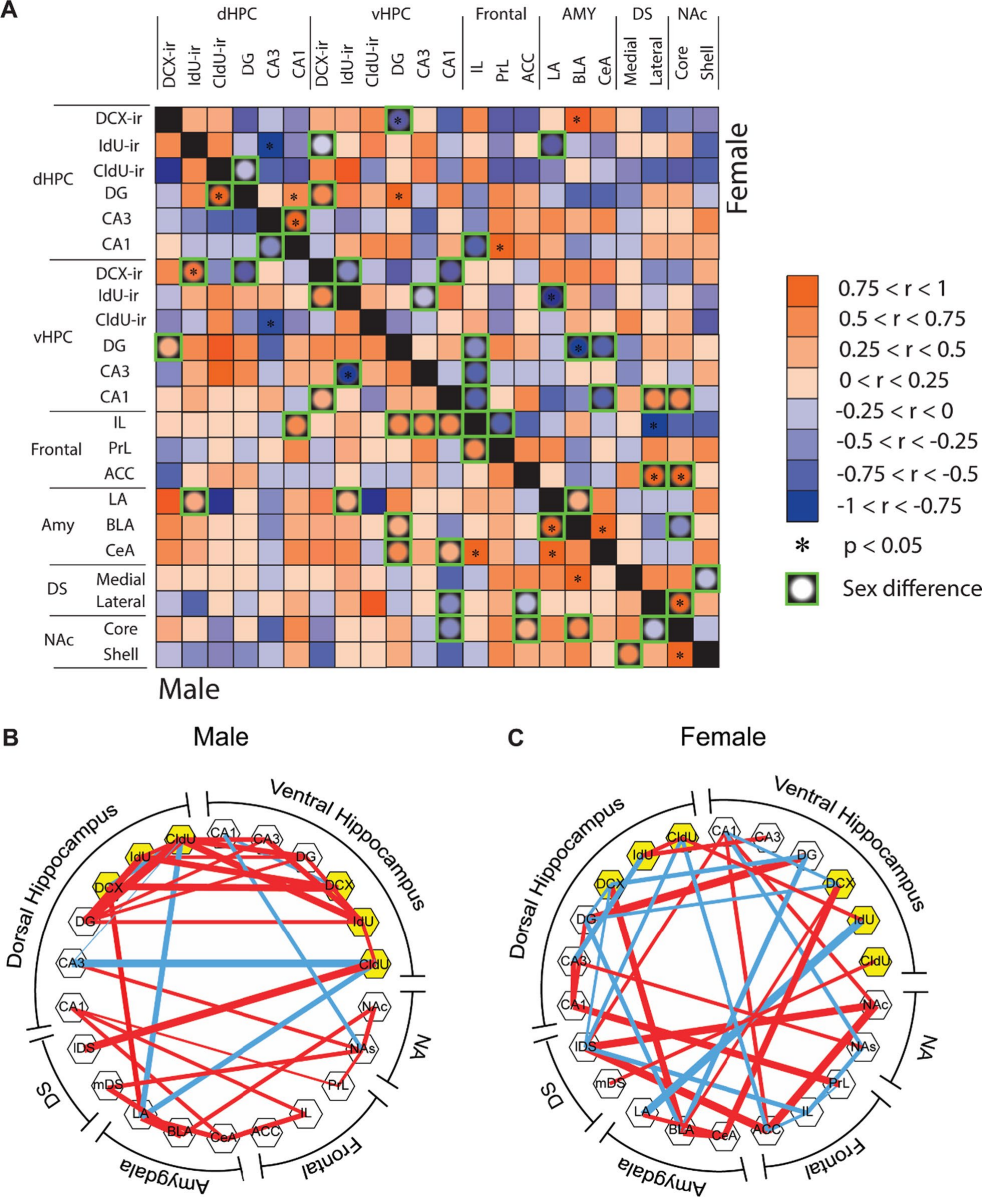
Sex differences in inter-regional correlations of zif268-ir cell density. **A** A heatmap showing correlation coefficients (*r*) of the density of zif268-ir cells between each brain region in males and females. Males and females showed distinct patterns of significant inter-regional correlations of zif268-ir cell density. * Indicates significant correlations (*p* < 0.05) and green boxes indicate sex differences between the correlations (*p* < 0.05). **B** Brain network maps were generated with correlations with coefficients larger than 0.67 or smaller than − 0.67 in males (left) and females (right) with *p* < 0.1. Red lines indicate positive correlations with wider lines indicating larger coefficients and blue lines indicate negative correlations with wider lines indicating smaller coefficients

Pearson product–moment correlations were calculated with the density of zif268-ir cells between 16 brain regions and six different populations of adult-born cells (double-labeled with zif268 and DCX, IdU, or CldU in the dorsal or ventral DG) to examine functional connectivity between these regions to activated new neurons of different ages (see Fig. 5A). As can be seen in Fig. 5B and C, there were mainly positive correlations between activation of new neurons of different ages within the hippocampus in males (19), with much fewer seen in females (4). In addition, there were more correlations of activated new neurons with regions outside the hippocampus in females (7, with only 2 significant to the amygdala) than in males (4 with none significant) (see Additional file 1: Table S1 for the detailed statistical data). The Fisher *z*-test statistic revealed significant sex differences in the 27 inter-regional correlations (see Additional file 1: Results for the detailed statistical data).

### Principal component analyses on the density of zif268-ir cells

**Fig. 6:**
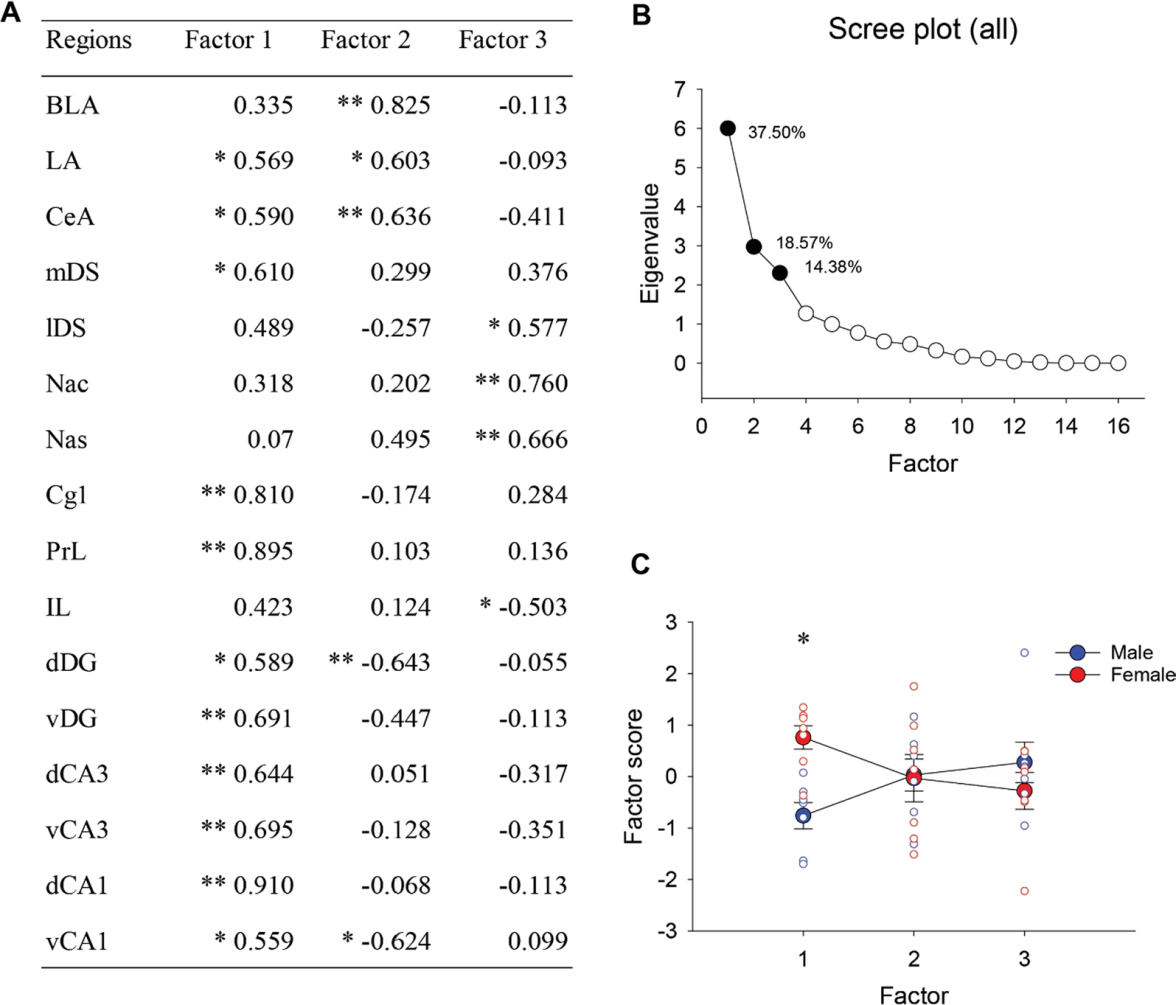
**A**–**C** Sex difference in the principal component analysis (PCA). Factor coordinates of the variables in the first three factors identified using principal component analysis (PCA) for zif268 activation (**A**). Scree plot for eigenvalues with the total percentage of variance for the first three factors (**B**). Eigenvalues for the first three factors were significant based on Horn’s parallel analysis. The first three factors explained 70.45% of the variances. A graph showing sex difference in the factor scores of individual samples (**C**). ANOVA and post hoc revealed males and females showed significant sex difference in the first factor. *Indicates *p* < 0.05 and ** indicates *p* < 0.01

Principal component analyses (PCA) were conducted with the density of zif268-ir cells in the 16 brain regions. PCA demonstrated that the first three principal components factors accounted for 70.45% of the variance with PC1 explaining 37.50% of the variance, PC2 explaining 18.57% and PC3 explaining 14.38% of the variance (see Fig. 6B). PC1 included significant positive loading on the density of zif268-ir cells in all of the hippocampus, most of the frontal cortex and the amygdala (except the IL and BLA) and included the mDS (see Fig. 6A). A repeated-measures ANOVA on the principal component scores revealed that females showed significantly greater positive scores compared to males in PC1 [interaction effect of sex by factor: *F* (2, 24) = 9.11, *p* = 0.001; post hoc: *p* = 0.031; see Fig. 6C], indicating that females had greater activation of zif268 among these regions compared to males.

## Discussion

We found that female rats were better at contextual pattern separation and had greater neural activation in the frontal cortex, dorsal CA1 region and in adult-born DGCs, in response to fear memory compared to males. Furthermore, we found distinct sex differences in functional connectivity in both direction (positive, negative) and in activation patterns between limbic regions, as males had more positive correlations among these regions than females. Intriguingly, we saw that activation of new neurons of different ages was intercorrelated among new neurons and with different regions in the hippocampus in males but not females. However, females, but not males, showed significant correlations between activated new neurons and the amygdala during fear memory retrieval. These results demonstrate that males and females employ different brain networks during fear memory retrieval. These findings highlight the importance of studying sex differences in fear memory and the contribution of adult neurogenesis to the neuronal network. They also have implications for targeting treatment of fear-related disorders between males and females.

### Females are better at fear-associated contextual pattern separation compared to males

Females showed greater discrimination between the two contexts compared to males on the last two trials days using the discrimination index. This is consistent with previous studies that reported a female advantage in fear-conditioning context discrimination tasks in rodents [11, 12] and in performance in emotional episodic memory tasks in humans [48–50]. However, others have found the opposite, with a male advantage in fear-conditioning context discrimination that used different spatial configurations for the contexts [51]. Indeed, in our own previous work, we found a male advantage in the ability for spatial pattern separation in rats [10] that was observed only among rats that rely more on allocentric (geometric) spatial cues [10]. The inconsistency between findings across studies may be due to sex differences in learning strategies and the types of cues (allo- or ego-centric) between the paradigms. Males rely preferentially on spatial strategies whereas females rely more idiothetic strategies in human and rodents [17, 52–54], which may explain why a male advantage was found when using different geometry (or shapes) of the conditioning chambers [51]. In contrast, the two contexts in the present study shared the same geometric cues, making it more difficult for rats relying on geometric cues to discriminate between the two contexts. Together these results suggest that males and females process contextual information differently and/or males and females rely on different learning strategies during a fear-conditioning context discrimination task so that the availability of favored memory cues influences their performance during a given task [55, 56].

In addition to the potential sex differences in learning strategies, the sex differences favoring females in the present contextual pattern separation task may be due to learned context discrimination. In our protocol, we gave 12 days of continual exposure to the two contexts but other protocols using fear context discrimination or generalization of fear use fewer trials [51, 57] which may also affect the results. In addition, previously learned memory can interfere with learning of new memories (reviewed in [58]) and intriguingly, neurogenesis minimizes proactive interference [59]. Indeed, pre-exposure to a conditioning context enhances the ability for contextual discrimination in females, but not in males [51]. Therefore, it is also plausible that previous experience (foot shocks) in the other context may affect the ability for contextual discrimination differently between the sexes in the present study. Further research is needed to determine whether there are sex differences in strategy use during a contextual learning, and to determine what types of memory cues and protocol differences might contribute to the sex difference in the ability for contextual pattern separation.

### Females show greater neuronal activation of young granule cells in the dorsal DG compared to males

We found that 3-week-old adult-born DGCs showed greater neuronal activation in females compared to males in response to fear memory retrieval. Our previous work demonstrates that adult-born DGCs in male rats mature faster than in females [20]. Therefore, it is possible that female 3-week-old adult-born DGCs are more immature and highly excitable in response to fear memory retrieval compared to males, however then we might have expected to see a sex difference favoring males in activation of the mostly younger DCX-ir cells which was not the case. Another possible explanation for the sex difference in neural activation of 3-week-old adult-born DGCs is that pattern separation circuits are differently recruited during re-exposure to familiar environment in female compared to male rats. Indeed, we see sex differences in the pattern and direction of correlations of activated new neurons in our study. Females show a coordinated activation of new neurons with the amygdala (and other regions), whereas males show more intercorrelations between different aged new neurons within the hippocampus. Previous studies demonstrated that adult-born DGCs play different roles depending on the age of DGCs, as younger DGCs play a role for pattern separation whereas older DGCs play a role for pattern completion [8, 60]. Therefore, greater neural activation of younger DGCs in females during memory retrieval may indeed be the reason for superior pattern separation performance by female rats compared to male rats in this task.

In addition to 3-week-old DGCs, we found sex differences in neural activation of 4-week-old DGCs depending on its location along the longitudinal axis. Females exhibited greater neural activation of 4-week-old adult-born DGCs in the dorsal DG whereas males exhibited greater neural activation of 4-week-old adult-born DGCs in the ventral DG compared to the opposite sex. This result suggests that 4-week-old adult-born DGCs play different functional roles in the dorsal and ventral DG between males and females, or that males and females differently recruit 4-week-old adult-born DGCs in the dorsal and ventral DG during contextual fear-conditioning paradigms. The dorsal hippocampus plays an important role for spatial learning and memory, and the ventral hippocampus is important for regulation of stress [14, 15, 61, 62]. However, sex differences in the contribution of adult-born DGCs depending on its location along the longitudinal axis and depending on maturity of DGCs to the hippocampal cognition have yet to be determined.

### Females show greater neuronal activation in the frontal cortex and dorsal CA1 in response to fear memory retrieval

We found that females, compared to males, showed greater neural activation in the frontal cortex despite there being no significant sex difference in fear memory during the activation trial. Previous studies have demonstrated that females have greater reliance on the frontal cortex (PrL, IL) to auditory fear memory acquisition, extinction and recall [63–66]. Collectively, these studies suggest that females rely on the frontal cortex to maintain fear memory.

Females also showed greater neural activation in the dorsal CA1 in response to fear memory than in males, consistent with other studies in contextual fear retrieval [67]. The CA1 in the hippocampus plays important roles for pattern completion during memory retrieval [68]. Further research is warranted to elucidate sex differences in the functional roles of dorsal CA1 during various memory tasks.

### Males and females show distinct patterns of functional connectivity between the frontal cortex, hippocampus and amygdala

The present study indicates significant sex differences in functional connectivity between the amygdala (LA, CeA), hippocampus (all subregions), dorsal striatum (mDS) and frontal cortex (PrL, ACC) where females show stronger positive connectivity among the regions in response to fear memory retrieval. This finding is consistent with resting-state functional connectivity and BOLD-signal changes in response to fear conditioning in humans [23, 69, 70] and with functional connectivity in rats [71]. Human females have greater resting-state functional connectivity between the amygdala, frontal regions and the hippocampus than human males [23, 69] and show greater BOLD-signal changes in the amygdala, and anterior cingulate cortex compared to human males to fear-conditioned stimuli [70]. Furthermore, we found sex differences in patterns of associations between neural activation in adult-born DGCs and neural activation in other brain regions, with females showing correlations to the amygdala that were not seen in males. To our knowledge, this is the first study demonstrating sex differences in functional connectivity of adult-born DGCs and other brain regions.

### Perspectives and significance

The present study found that female rats acquired pattern discrimination faster than males in a contextual pattern separation task. However, despite similar fear memory, females showed greater activation of new neurons in the dorsal dentate gyrus in response to fear memory. Furthermore, males and females showed distinct functional connectivity between limbic regions and activated new neurons during fear memory retrieval, with more correlations between activated new neurons of different ages in males but more correlations with activated new neurons to other limbic regions in females. These data suggest that the functional contribution of adult neurogenesis to pattern separation and pattern completion may be via different pathways in males and females. To our knowledge, the dynamics of the functional connectivity with activated new neurons have not been recorded previously and the functional significance of these sex differences remains to be determined. Future studies manipulating the activity of adult-born neurons in the dentate gyrus are needed to determine how adult-born young neurons contribute to the functional connectivity of long-term fear memory. Furthermore, ovarian hormones may play an important role for modulating the sex difference in prevalence of PTSD as postmenopausal females show decreased prevalence of PTSD [72]. Therefore, further studies examining how estradiol and age modulate the ability for pattern separation are needed in part to help elucidate the mechanisms underlying sex differences in PTSD.

## Conclusion

Our data demonstrate that females, compared to males, show greater context discrimination, greater activation of 3-week-old adult-born DGCs in response to memory retrieval, and strong functional connectivity in the frontal cortex, the hippocampus, dorsal striatum and the amygdala during fear memory retrieval. Our findings indicate that sex differences exist in the underlying neural mechanisms and network activation even when no significant sex difference is observed in fear memory retrieval. Our work highlights the importance of elucidating sex-specific neural connections that may contribute to differences in susceptibility to fear-related disorders, such as PTSD. It also underscores that any treatments for fear-related disorders will need to consider sex as very different neural mechanisms may be underlying fear memory.

## Supplementary Information

**Additional file 1: Table S1.** A list of antibodies used in the present study. **Table S2.** A list of reagents used in the present study. **Table S3.** Mean (±SEM) duration of rearing, grooming and non-specific behaviors in males and females during the activation trial. **Figure S1.** A-B: Mean (±SEM) density of adult-born cells in the dorsal (A) and ventral (B) dentate gyrus. There were no significant sex differences in the density of DCX-ir cells, IdU-ir cells or CldU-ir cells in the dorsal or ventral dentate gyrus. **Figure S2**. A-C: Mean (±SEM) density of zif268-ir cells in the nucleus accumbens (A), the amygdala (B) and the dorsal striatum (C). The density of zif268-ir cells in the nucleus accumbens shell is greater compared to the nucleus accumbens core (A). Females, compared to males, showed greater density of zif268-ir cells in the amygdala (B). A priori we found that the density of zif268-ir cells was significantly greater in the lDS compared to mDS in males, but not in females. * indicates p < 0.05.

## Data Availability

The datasets used and/or analyzed during the current study are available from the corresponding author on reasonable request.

## References

[1] Kessler RC, Petukhova M, Sampson NA, Zaslavsky AM, Wittchen H-U (2012). Twelve-month and lifetime prevalence and lifetime morbid risk of anxiety and mood disorders in the United States. Int J Methods Psychiatr Res.

[2] Kessler RC, Sonnega A, Nelson CB, Bromet E (1995). Posttraumatic stress disorder in the national comorbidity survey. Arch Gen Psychiatryatry.

[3] Campbell S, Marriott M, Nahmias C, MacQueen GM (2004). Lower hippocampal volume in patients suffering from depression: a meta-analysis. Am J Psychiatry.

[4] O’Doherty DCM, Chitty KM, Saddiqui S, Bennett MR, Lagopoulos J (2015). A systematic review and meta-analysis of magnetic resonance imaging measurement of structural volumes in posttraumatic stress disorder. Psychiatry Res Neuroimaging.

[5] Marr D (1971). Simple memory: a theory for archicortex. Philos Trans R Soc London Ser B, Biol Sci.

[6] Yassa MA, Stark CEL (2011). Pattern separation in the hippocampus. Trends Neurosci.

[7] Lange I, Goossens L, Michielse S, Bakker J, Lissek S, Papalini S (2017). Behavioral pattern separation and its link to the neural mechanisms of fear generalization. Soc Cogn Affect Neurosci.

[8] Clelland CD, Choi M, Romberg C, Clemenson GD, Fragniere A, Tyers P (2009). A functional role for adult hippocampal neurogenesis in spatial pattern separation. Science (80)..

[9] Anacker C, Luna VM, Stevens GS, Millette A, Shores R, Jimenez JC (2018). Hippocampal neurogenesis confers stress resilience by inhibiting the ventral dentate gyrus. Nature.

[10] Yagi S, Chow C, Lieblich SE, Galea LAM (2016). Sex and strategy use matters for pattern separation, adult neurogenesis, and immediate early gene expression in the hippocampus. Hippocampus.

[11] Foilb AR, Bals J, Sarlitto MC, Christianson JP (2018). Sex differences in fear discrimination do not manifest as differences in conditioned inhibition. Learn Mem.

[12] Day HLL, Reed MM, Stevenson CW (2016). Neurobiology of learning and memory sex differences in discriminating between cues predicting threat and safety. Neurobiol Learn Mem.

[13] Snyder JS, Ferrante SC, Cameron HA (2012). Late maturation of adult-born neurons in the temporal dentate gyrus. PLoS ONE.

[14] Moser E, Moser MB, Andersen P (1993). Spatial learning impairment parallels the magnitude of dorsal hippocampal lesions, but is hardly present following ventral lesions. J Neurosci.

[15] Kjelstrup KG, Tuvnes FA, Steffenach H-A, Murison R, Moser EI, Moser M-B (2002). Reduced fear expression after lesions of the ventral hippocampus. Proc Natl Acad Sci U S A.

[16] Galea LAM, Kimura D (1993). Sex differences in route-learning. Pers Individ Dif.

[17] Williams CL, Barnett AM, Meek WH (1990). Organizational effects of early gonadal secretions on sexual differentiation in spatial memory. Behav Neurosci.

[18] Nakashiba T, Young JZ, Mchugh TJ, Buhl DL, Nakashiba T, Young JZ (2008). Hippocampal learning transgenic inhibition of synaptic transmission reveals role of CA3 output in hippocampal learning. Science (80)..

[19] Chow C, Epp JR, Lieblich SE, Barha CK, Galea LAM (2013). Sex differences in neurogenesis and activation of new neurons in response to spatial learning and memory. Psychoneuroendocrinology.

[20] Yagi S, Splinter JEJ, Tai D, Wong S, Wen Y, Galea LAM (2020). Sex differences in maturation and attrition of adult neurogenesis in the hippocampus. eNeuro..

[21] Hermans EJ, Kanen JW, Tambini A, Fernández G, Davachi L, Phelps EA (2017). Persistence of amygdala-hippocampal connectivity and multi-voxel correlation structures during awake rest after fear learning predicts long-term expression of fear. Cereb Cortex.

[22] Shvil E, Rusch HL, Sullivan GM, Neria Y (2013). Neural, psychophysiological, and behavioral markers of fear processing in PTSD: a review of the literature. Curr Psychiatry Rep.

[23] Engman J, Linnman C, Van Dijk KRA, Milad MR (2016). Amygdala subnuclei resting-state functional connectivity sex and estrogen differences. Psychoneuroendocrinology.

[24] Tanimizu T, Kenney JW, Okano E, Kadoma K, Frankland PW, Kida S (2017). Functional connectivity of multiple brain regions required for the consolidation of social recognition memory. J Neurosci.

[25] Wheeler AL, Teixeira CM, Wang AH, Xiong X, Kovacevic N, Lerch JP, et al. Identification of a functional connectome for long-term fear memory in mice. PLoS Comput Biol. 2013;9. 10.1371/journal.pcbi.1002853 PMC3536620 23300432

[26] Guzowski JF, Lyford GL, Stevenson GD, Houston FP, McGaugh JL, Worley PF (2000). Inhibition of activity-dependent arc protein expression in the rat hippocampus impairs the maintenance of long-term potentiation and the consolidation of long-term memory. J Neurosci.

[27] Guzowski JF, Setlow B, Wagner EK, McGaugh JL (2001). Experience-dependent gene expression in the rat hippocampus after spatial learning: a comparison of the immediate-early genes Arc, c-fos, and zif268. J Neurosci.

[28] Jones MW, Errington ML, French PJ, Fine A, Bliss TV, Garel S (2001). A requirement for the immediate early gene Zif268 in the expression of late LTP and long-term memories. Nat Neurosci.

[29] Huckleberry KA, Shue F, Copeland T, Chitwood RA, Yin W, Drew MR (2018). Dorsal and ventral hippocampal adult-born neurons contribute to context fear memory. Neuropsychopharmacology.

[30] Nakashiba T, Young JZ, Mchugh TJ, Buhl DL, Nakashiba T, Young JZ (2018). Hippocampal learning transgenic inhibition of synaptic transmission reveals role of CA3 output in hippocampal learning. Science.

[31] Snyder JS, Choe JS, Clifford MA, Jeurling SI, Hurley P, Brown A (2009). Adult-born hippocampal neurons are more numerous, faster maturing, and more involved in behavior in rats than in mice. J Neurosci.

[32] Llorens-Martín M, Trejo JL (2011). Mifepristone prevents stress-induced apoptosis in newborn neurons and increases ampa receptor expression in the dentate gyrus of c57/bl6 mice. PLoS ONE.

[33] Leuner B, Glasper ER, Gould E (2009). Thymidine analog methods for studies of adult neurogenesis are not equally sensitive. Biosystems.

[34] Kee N, Si S, Boonstra R, Wojtowicz JM (2002). The utility of Ki-67 and BrdU as proliferative markers of adult neurogenesis. J Neurosci Methods.

[35] Miller I, Min M, Yang C, Tian C, Gookin S, Carter D (2018). Ki67 is a graded rather than a binary marker of proliferation versus quiescence. Cell Rep.

[36] Mchugh TJ, Jones MW, Quinn JJ, Balthasar N, Coppari R, Elmquist JK (2007). Dentate gyrus NMDA receptors mediate rapid pattern separation in the hippocampal network. Science (80)..

[37] Gruene TM, Flick K, Stefano A, Shea SD, Shansky RM, Gruene BTM (2015). Sexually divergent expression of active and passive conditioned fear responses in rats. Elife.

[38] Brown JP, Couillard-Després S, Cooper-Kuhn CM, Winkler J, Aigner L, Kuhn HG (2003). Transient expression of doublecortin during adult neurogenesis. J Comp Neurol.

[39] Tuttle AH, Rankin MM, Teta M, Sartori DJ, Stein GM, Kim GJ, Virgilio C, Granger A, Zhou D, Long SH, Schiffman AB, Kushner JA (2010). Immunofluorescent detection of two thymidine analogues (CldU and IdU) in primary tissue. J Vis Exp..

[40] Gundersen HJ, Jensen EB (1987). The efficiency of systematic sampling in stereology and its prediction. J Microsc.

[41] Paxinos G, Watson C (2005). The rat brain in stereotaxic coordinates.

[42] Banasr M, Soumier A, Hery M, Mocaër E, Daszuta A (2006). Agomelatine, a new antidepressant, induces regional changes in hippocampal neurogenesis. Biol Psychiatry.

[43] Fanselow MS, Dong HW (2010). Are the dorsal and ventral hippocampus functionally distinct structures?. Neuron.

[44] Hubscher CH, Brooks DL, Johnson JR (2005). A quantitative method for assessing stages of the rat estrous cycle. Biotech Histochem.

[45] Warren SG, Humphreys AG, Juraska JM, Greenough WT (1995). LTP varies across the estrous cycle: enhanced synaptic plasticity in proestrus rats. Brain Res.

[46] Yagi S, Drewczynski D, Wainwright SR, Barha CK, Hershorn O, Galea LAM (2017). Sex and estrous cycle differences in immediate early gene activation in the hippocampus and the dorsal striatum after the cue competition task. Horm Behav.

[47] Franklin SB, Gibson DJ, Robertson PA, Pohlmann JT, Fralish JS (1995). Parallel analysis: a method for determining significant principal components. J Veg Sci.

[48] Naveh-benjamin M, Guez J, Kilb A, Reedy S (2004). The associative memory deficit of older adults : further support using face-name associations. Psychol Aging.

[49] Gavazzeni J, Andersson T, Ba L, Wiens S (2012). Age, gender, and arousal in recognition of negative and neutral pictures 1 year later. Psychol Aging.

[50] Andreano JM, Cahill L (2009). Sex influences on the neurobiology of learning and memory. Learn Mem.

[51] Keiser AA, Turnbull LM, Darian MA, Feldman DE, Song I, Tronson NC (2017). Sex differences in context fear generalization and recruitment of hippocampus and amygdala during retrieval. Neuropsychopharmacology.

[52] Sandstrom NJ, Kaufman J, Huettel SA (1998). Males and females use different distal cues in a virtual environment navigation task 1. Cogn Brain Res.

[53] Barkley CL, Gabriel KI (2007). Sex differences in cue perception in a visual scene: investigation of cue type. Behav Neurosci.

[54] Chai XJ, Jacobs LF (2010). Effects of cue types on sex differences in human spatial memory. Behav Brain Res.

[55] Chen CS, Knep E, Han A, Ebitz RB, Grissom NM (2021). Sex differences in learning from exploration. Elife.

[56] Tronson NC (2018). Focus on females: a less biased approach for studying strategies and mechanisms of memory. Curr Opin Behav Sci.

[57] Lynch J, Cullen PK, Jasnow AM, Riccio DC (2013). Sex differences in the generalization of fear as a function of retention intervals. Learn Mem.

[58] Yassa MA, Reagh ZM (2013). Competitive trace theory: a role for the hippocampus in contextual interference during retrieval. Front Behav Neurosci.

[59] Epp JR, Silva Mera R, Köhler S, Josselyn SA, Frankland PW (2016). Neurogenesis-mediated forgetting minimizes proactive interference. Nat Commun.

[60] Nakashiba T, Cushman JD, Pelkey KA, Renaudineau S, Buhl DL, McHugh TJ (2012). Young dentate granule cells mediate pattern separation, whereas old granule cells facilitate pattern completion. Cell.

[61] Henke PG (1990). Hippocampal pathway to the amygdala and stress ulcer development. Brain Res Bull.

[62] Pothuizen HHJ, Zhang W-N, Jongen-Rêlo AL, Feldon J, Yee BK (2004). Dissociation of function between the dorsal and the ventral hippocampus in spatial learning abilities of the rat: a within-subject, within- task comparison of reference and working. Dissociation of function between the dorsal and the ventral hippocam. Eur J Neurosci.

[63] Fenton GE, Pollard AK, Halliday DM, Mason R, Bredy TW, Stevenson CW (2014). Persistent prelimbic cortex activity contributes to enhanced learned fear expression in females. Learn Mem.

[64] Fenton GE, Halliday DM, Mason R, Bredy TW, Stevenson CW (2016). Sex differences in learned fear expression and extinction involve altered gamma oscillations in medial prefrontal cortex. Neurobiol Learn Mem.

[65] Baran SE, Armstrong CE, Niren DC, Conrad CD (2010). Prefrontal cortex lesions and sex differences in fear extinction and perseveration. Learn Mem.

[66] Kirry AJ, Durigan DJ, Twining RC, Gilmartin MR (2019). Estrous cycle stage gates sex differences in prefrontal muscarinic control of fear memory formation. Neurobiol Learn Mem.

[67] Colon LM, Poulos AM (2020). Contextual processing elicits sex differences in dorsal hippocampus activation following footshock and context fear retrieval. Behav Brain Res.

[68] Hunsaker MR, Kesner RP (2013). The operation of pattern separation and pattern completion processes associated with different attributes or domains of memory. Neurosci Biobehav Rev.

[69] Kogler L, Müller VI, Seidel EM, Boubela R, Kalcher K, Moser E (2016). Sex differences in the functional connectivity of the amygdalae in association with cortisol. Neuroimage.

[70] Lebron-Milad K, Abbs B, Milad MR, Linnman C, Rougemount-Bücking A, Zeidan MA (2012). Sex differences in the neurobiology of fear conditioning and extinction: a preliminary fMRI study of shared sex differences with stress-arousal circuitry. Biol Mood Anxiety Disord.

[71] Worley NB, Everett SR, Foilb AR, Christianson JP (2020). Functional networks activated by controllable and uncontrollable stress in male and female rats. Neurobiol Stress.

[72] Creamer M, Parslow R (2008). Trauma exposure and posttraumatic stress disorder in the elderly: a community prevalence study. Am J Geriatr Psychiatry.

