# Supplementary material for: Sex differences in contextual pattern separation, neurogenesis, and functional connectivity within the limbic system
Source: Biol Sex Differ. 2022 Jul 23;13:42. doi: 10.1186/s13293-022-00450-2 (PMC9308289; doi:10.1186/s13293-022-00450-2)
Supplement: Supplementary file 1 — Additional file 1: Table S1. A list of antibodies used in the present study. Table S2. A list of reagents used in the present study. Table S3. Mean (±SEM) duration of rearing, grooming and non-specific behaviors in males and females during the activation trial. Figure S1. A-B: Mean (±SEM) density of adult-born cells in the dorsal (A) and ventral (B) dentate gyrus. There were no significant sex differences in the density of DCX-ir cells, IdU-ir cells or CldU-ir cells in the dorsal or ventral dentate gyrus. Figure S2. A-C: Mean (±SEM) density of zif268-ir cells in the nucleus accumbens (A), the amygdala (B) and the dorsal striatum (C). The density of zif268-ir cells in the nucleus accumbens shell is greater compared to the nucleus accumbens core (A). Females, compared to males, showed greater density of zif268-ir cells in the amygdala (B). A priori we found that the density of zif268-ir cells was significantly greater in the lDS compared to mDS in males, but not in females. * indicates p < 0.05. [file 13293_2022_450_MOESM1_ESM.docx]

**Additional Material**

| Table S1: A list of antibodies used in the present study | | | |
| --- | --- | --- | --- |
| Product | Host Species | Manufacturer | Catalog # |
| Anti-BrdU antibody [BU1/75] | rat | Abcam | ab6326 |
| Anti-BrdU antibody [B44] | mouse | BD Biosciences | 347580 |
| Anti-doublecortin | goat | Santa Cruz | sc-8066 |
| Anti-EGR1/zif268 | rabbit | Santa Cruz | sc-189 |
| Anti-mouse Alexa Fluor 488 | Donkey | Invitrogen | A21203 |
| Anti-rat Alexa Fluor 594 | Donkey | Invitrogen | A21209 |
| Anti-goat Alexa Fluor 488 | Donkey | Invitrogen | A11055 |
| Anti-rabbit Alexa Fluor 594 | Donkey | Invitrogen | A21207 |
| Anti-rabbit Alexa Fluor 647 | Donkey | Jackson ImmunoResearch | 711-605-152 |

| Table S2: A list of reagents used in the present study | | |
| --- | --- | --- |
| Product | Manufacturer | Catalog # |
| 5-chloro-2'-deoxy-uridine | Cayman Chemical | 50-90-8 |
| 5 Iodo 2' deoxyuridine | MP Biomedicals | 210035705 |
| Triton X-100 | Sigma-Aldrich | T8787-250ML |
| Trizma hydrochloride | Sigma-Aldrich | T3253-1KG |
| Trizma base | Sigma-Aldrich | T6066-500G |
| Hydrochrolic acid | Fisher Scientific | SA431-500ML |
| Sodium Chrolide | Sigma-Aldrich | S9625-1KG |
| Tween 20 | Sigma-Aldrich | P9416-50ML |
| Polyvinylalcohol | Sigma-Aldrich | P8136-250G |
| DABCO 33LV | Sigma-Aldrich | 290734-100ML |

| Table S3: Mean (±SEM) duration of rearing, grooming and non-specific behaviors in males and females during the activation trial. There were no significant sex differences in any of the behaviors (p > 0.889). | | | |
| --- | --- | --- | --- |
|  | Behaviors (sec) | | |
|  | Rearing | Grooming | Other |
| Male | 4.38±2.03 | 0 | 54.5±8.81 |
| Female | 3.38±1.51 | 1.50±1.13 | 55.75±16.08 |


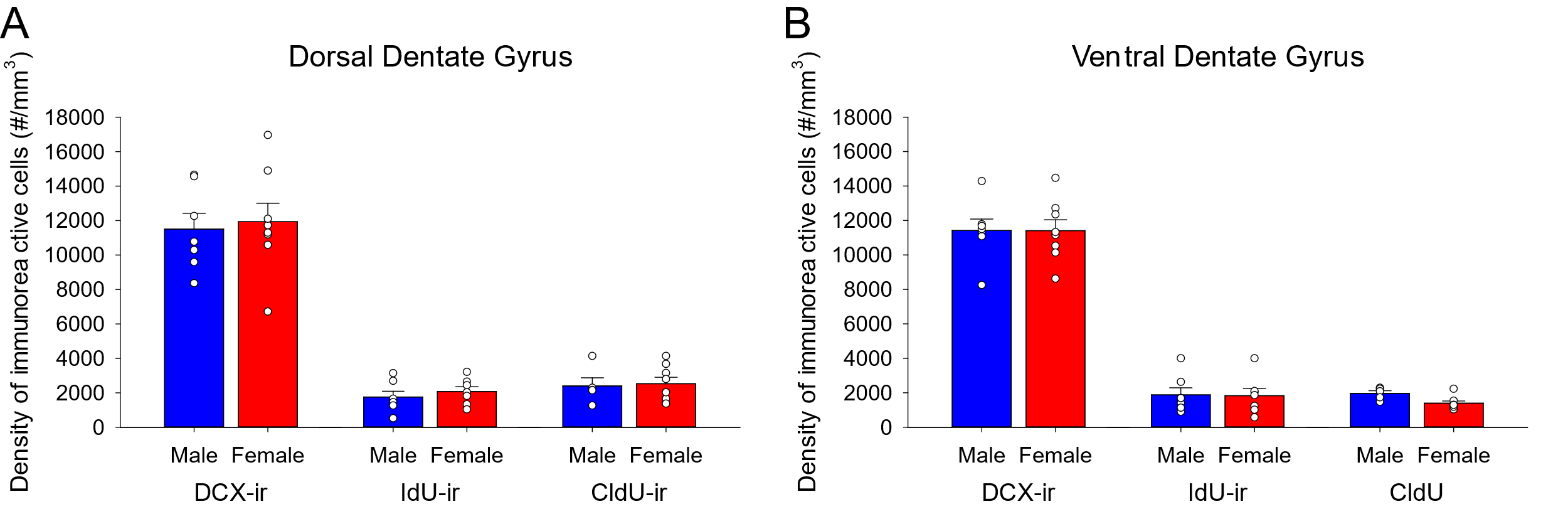


**Figure S1. A-B: Mean (±SEM) density of adult-born cells in the dorsal (A) and ventral (B) dentate gyrus.** There were no significant sex differences in the density of DCX-ir cells, IdU-ir cells or CldU-ir cells in the dorsal or ventral dentate gyrus.


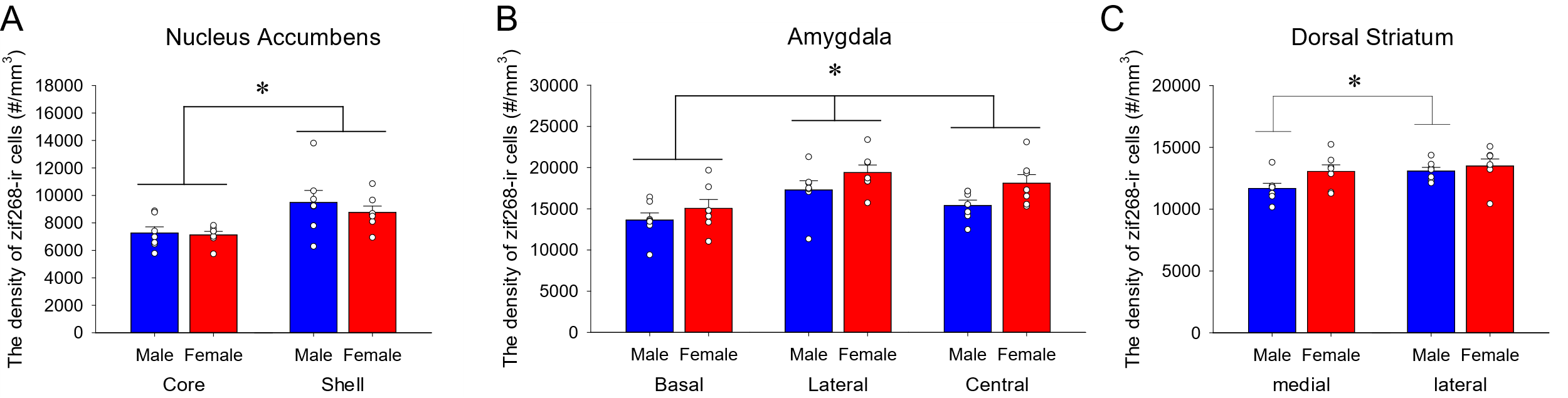


**Figure S2. A-C: Mean (±SEM) density of zif268-ir cells in the nucleus accumbens (A), the amygdala (B) and the dorsal striatum (C).** The density of zif268-ir cells in the nucleus accumbens shell is greater compared to the nucleus accumbens core (A). Females, compared to males, showed greater density of zif268-ir cells in the amygdala (B). A priori we found that the density of zif268-ir cells was significantly greater in the lDS compared to mDS in males, but not in females. * indicates p < 0.05.

Additional Results

Inter-regional Correlations

In males, within the hippocampus, there were significant correlations between activation of new neurons and activation of subregions of the hippocampus between vDG IdU/zif268-ir cells and vCA3 zif268-ir cells [r(7) = 0.801, p = 0.030], between dDG CldU/zif268-ir cells and dDG zif268-ir cells [r(5) = 0.9926, p = 0.001], and between vDG CldU/zif268-ir cells and dCA3 zif268-ir cells [r(5) = -0.915, p = 0.029]. In females, within the hippocampus, there were significant correlations between the density of dDG IdU/zif268-ir cells and either the vDG DCX/zif268-ir cells [r(7) = 0.848, p = 0.016] or the dCA3 zif268-ir cells [r(7) = -0.794, p = 0.033] and between the density of dDG DCX/zif268-ir cells and the vDG zif268-ir cells [r(8) = -0.745, p = 0.034]. Within females there were also significant correlations between activated new neurons and brain regions outside of the hippocampus (that did not exist in males) between dDG DCX/zif268-ir cells and BLA zif268-ir cells [r(7) = 0.860, p = 0.013] and between vDG IdU/zif268-ir cells and LA zif268-ir cells [r(6) = -0.936, p = 0.006].

Correlations between the 15 different brain regions in males were between the BLA and LA [r(7) = 0.922, p = 0.003], between BLA and mDS [r(7) = 0.789, p = 0.035], between CeA and LA [r(7) = 0.780, p = 0.039] between CeA and IL [r(7) = 0.779, p = 0.039], and between NAc and NAs [r(7) = 0.755, p = 0.050]. In females, between BLA and CeA [r(7) = 0.860, p = 0.013], between BLA and vDG [r(7) = -0.785, p = 0.037], between lDS and NAc [r(7) = 0.910, p = 0.004], between lDS and ACC [r(7) = 0.939, p = 0.002], between lDS and IL [r(7) = -0.760, p = 0.047], between ACC and NAc [r(7) = 0.953, p = 0.001], between PL and dCA1 [r(7) = 0.888, p = 0.008], between dDG and vDG [r(8) = 0.937, p = 0.001], between dDG and dCA1 [r(8) = 0.718, p = 0.045], and between dCA3 and dCA1 [r(8) = 0.790, p = 0.020].

Sex differences in inter-regional correlations

The Fischer z-test statistic revealed significant sex differences in correlations between activation of new neurons and activation of subregions of the hippocampus between the density of vDG IdU/zif268-ir cells and vDG DCX/zif268-ir cells [ Z(13) = 1.652, p = 0.049], between dDG DCX/zif268-ir cells and vDG zif268-ir cells [ Z (13) = 1.723, p = 0.042], vDG DCX/zif268-ir cells and dDG zif268-ir cells [ Z(14) = 2.427, p = 0.008 ], vDG DCX/zif268-ir cells and vDG zif268-ir cells [ Z(14) = 2.061, p = 0.020], vDG DCX/zif268-ir cells and vCA1 zif268-ir cells [ Z(14) = 1.995, p = 0.023], vDG IdU/zif268-ir cells and vCA3 zif268-ir cells [ Z(13) = 1.669, p = 0.048], and dDG CldU/zif268-ir cells and dDG zif268-ir cells [ Z(11) = 3.276, p = 0.001]. In addition, there were also significant correlations between activated new neurons and brain regions outside of the hippocampus, between dDG IdU/zif268-ir cells and LA zif268-ir cells [ Z(12) = 1.715, p = 0.043], vDG IdU/zif268-ir cells and LA zif268-ir cells [ Z(12) = 2.873, p = 0.002].

Furthermore, there were significant sex differences in the correlations between the 15 different brain regions including between LA and BLA [Z(13) = 1.724, p = 0.042], BLA and NAc [Z(13) = 1.765, p = 0.039], lDS and NAc [Z(13) = 2.177, p = 0.015], mDS and NAs [Z(13) = 1.701, p = 0.044], lDS and ACC [Z(13) = 2.696, p = 0.004], NAc and ACC [Z (13) = 2.089, p = 0.018], PL and IL [Z(13) = 2.184, p = 0.014], vDG and BLA [Z(13) = 2.115, p = 0.017], vDG and CeA [Z(13) = 2.133, p = 0.016], vDG and IL [Z(13) = 1.651, p = 0.049], vCA3 and IL [ Z(13) = 1.907, p = 0.028], dCA1 and IL [ Z (13) = 2.143, p = 0.016], dCA3 and dCA1 [ Z (14) = 2.202, p = 0.014], CeA and vCA1 [ z(13) = 1.748, p = 0.040], lDS and vCA1 [ Z(13) = 1.712, p = 0.043], NAc and vCA1 [ Z(13) = 1.967, p = 0.025], IL and vCA1 [ Z(13) = 1.881, p = 0.030].
